# Prognostic implication of bronchoalveolar lavage fluid analysis in patients with *Pneumocystis jirovecii* pneumonia without human immunodeficiency virus infection

**DOI:** 10.1186/s12890-022-02041-8

**Published:** 2022-06-26

**Authors:** Chiwook Chung, Chae Man Lim, Yeon-Mok Oh, Sang Bum Hong, Chang-Min Choi, Jin Won Huh, Sei Won Lee, Jae Seung Lee, Kyung-Wook Jo, Wonjun Ji, Chan-Jeoung Park, Mina Kim, Heungsup Sung, Young-Uk Cho, Hyo Sin Cho, Ho Cheol Kim

**Affiliations:** 1 Department of Pulmonary and Critical Care Medicine, Asan Medical Center, University of Ulsan College of Medicine, 88 Olympic-ro 43-gil, Songpa-gu, Seoul, 05505 Republic of Korea; 2 Department of Laboratory Medicine, Asan Medical Center, University of Ulsan College of Medicine, Seoul, Republic of Korea; 3 University of Ulsan College of Medicine, Seoul, Republic of Korea

**Keywords:** *Pneumocystis jirovecii*, Bronchoalveolar lavage, Lymphocyte, Mortality

## Abstract

**Background:**

The prognostic value of bronchoalveolar lavage (BAL) fluid analysis in non-human immunodeficiency virus (HIV)-infected patients with *Pneumocystis jirovecii* pneumonia (PJP) has not been well elucidated. We aimed to investigate the prognostic implication of BAL fluid analysis in non-HIV patients with PJP.

**Methods:**

The data of 178 non-HIV patients diagnosed with PJP based on the results of the polymerase chain reaction assay of BAL fluid specimens between April 2018 and December 2020 were retrospectively reviewed. The clinical characteristics, laboratory findings, and BAL fluid analysis results of patients who died within 90 days after hospital admission were compared.

**Results:**

Twenty patients (11.2%) died within 90 days from admission. The neutrophil count in BAL fluid was significantly higher (median 22.0%, interquartile range [IQR] 2.0–46.0% vs. median 6.0%, IQR 2.0–18.0%, *P* = 0.044), while the lymphocyte count was significantly lower (median 24.0%, IQR 7.0–37.0% vs. median 41.0%, IQR 22.5–60.5%, *P* = 0.001) in the non-survivor group compared with that in the survivor group. In the multivariate analysis, the C-reactive protein level (odds ratio [OR] 1.093, 95% confidence interval [CI] 1.020–1.170, *P* = 0.011) and a BAL fluid lymphocyte count of ≤ 30% (OR 3.353, 95% CI 1.101–10.216, *P* = 0.033) were independently associated with mortality after adjusting for albumin and lactate dehydrogenase levels.

**Conclusion:**

A low lymphocyte count in BAL fluid may be a predictor of mortality in non-HIV patients with PJP.

**Supplementary Information:**

The online version contains supplementary material available at 10.1186/s12890-022-02041-8.

## Background

*Pneumocystis jirovecii* pneumonia (PJP) is one of the most prevalent and lethal opportunistic infections in patients infected with human immunodeficiency virus (HIV) [1]. The incidence of PJP is increasing in patients without HIV infection but with immunocompromising conditions, including hematological malignancies, solid cancers, organ transplantations, and autoimmune diseases [2–4]. The treatment outcomes of HIV-infected patients with PJP have greatly improved after receiving antiretroviral therapy and adjunctive corticosteroids [1, 5]. However, in non-HIV-infected patients, the mortality of PJP remains high (> 30%) [3, 6, 7], and the role of adjunctive corticosteroid therapy is unclear [7–9]. Various clinical parameters are used to predict the prognosis of non-HIV-infected patients with PJP, including older age, female sex, respiratory failure, high lactate dehydrogenase (LD) and C-reactive protein (CRP) levels, low albumin level, lymphopenia, other co-infections, pneumothorax, and mechanical ventilation [6, 7, 9].

Polymerase chain reaction (PCR) assay of bronchoalveolar lavage (BAL) fluid has been considered the gold standard method for diagnosing PJP [1, 10]. Recent studies have shown the prognostic role of the cellular properties of BAL fluid, including neutrophilia and lymphopenia, in non-HIV patients with PJP [11, 12]. The cycle threshold (CT) value of the quantitative PCR test for *P. jirovecii* can be a surrogate marker of fungal burden, and some studies have reported the association between CT value and prognosis in non-HIV patients with PJP [9, 13]; however, these results have not been validated. Therefore, we aimed to evaluate the prognostic value of BAL fluid analysis in non-HIV patients with PJP.

## Methods

### Study population

Patients who developed symptoms (fever, cough, sputum, and dyspnea) and had radiological findings (ground-glass opacities, reticular opacities, septal thickening, consolidations, or pulmonary cysts on chest computed tomography) compatible with PJP and underwent fiberoptic bronchoscopy with BAL, and who were diagnosed with PJP through a PCR assay of the BAL fluid between April 2018 and December 2020 at Asan Medical Center, Seoul, Republic of Korea, were screened [1, 14]. In 2020, 29,764 patients were newly diagnosed with cancer, 192,775 received chemotherapy, 987 underwent solid organ transplantation, and 348 underwent hematopoietic stem cell transplantation in Asan Medical Center. Patients with a CT value of > 35 on quantitative PCR test for detection of *P. jirovecii* were considered to have colonization and thus excluded from this study [15]. Patients aged < 16 years or with confirmed HIV infection were also excluded. The study was approved by the Institutional Review Board of Asan Medical Center (approval no. 2021-0429). The requirement for informed consent was waived due to the retrospective nature of the study.

### Data collection and definitions

Clinical data, including demographics, comorbidities, laboratory findings, and BAL fluid analysis results, were retrospectively collected from the medical records. All available baseline clinical parameters were obtained within 5 days from the performance of initial bronchoscopy. The outcome was set as in-hospital 90-day all-cause mortality, which was defined as death from any cause occurring within 90 days from the day of admission. Severe PJP was defined as the occurrence of hypoxemia prior to the performance of bronchoscopy (a partial pressure of arterial oxygen [PaO_2_] while the patient is breathing room air of < 70 mmHg or an alveolar–arterial oxygen pressure gradient [(A-a)DO_2_] of > 35 mmHg) [1].

### BAL fluid analysis and PCR test for *P. jirovecii*

BAL was performed during bronchoscopy using a fiberoptic cable based on the standard protocol [16]. A hemocytometer was used to measure the total cell count. A BAL fluid sample of 10^3^ cells was centrifuged at 500 rpm for 5 min at room temperature. Differential cell count was performed to determine the neutrophil, lymphocyte, eosinophil, and alveolar macrophage counts [11, 17]. DNA was extracted from the BAL fluid using a QIAamp DNA Mini Kit (Qiagen, Valencia, CA, USA) according to the manufacturer’s instructions. After centrifuging the BAL fluid sample at 3000 rpm for 5 min, a standardized volume of 2 mL was extracted. The elution volume was 100 μL. The AmpliSens *P. jirovecii* (*carinii*)-FRT PCR kit (AmpliSens, Bratislava, Slovak Republic) was used for the semi-quantitative measurement of *P. jirovecii* DNA according to the manufacturer’s instructions. The result was reported as the CT value from real-time PCR, defined as the cycle number at which the fluorescence generated within a reaction crossed the fluorescence threshold [15].

### Statistical analysis

Continuous variables are expressed as mean (standard deviation [SD]) or median (interquartile range [IQR]), and categorical variables are expressed as numbers (percentage). The Student’s *t*-test or the Mann–Whitney test was used to compare continuous variables. The χ^2^ or Fisher’s exact test was used to compare categorical variables. Receiver operating characteristic curve analysis was used to evaluate the diagnostic values of the cellular properties of BAL fluid. The optimal cutoff value was calculated using the Youden index. Logistic regression was used to identify the risk factors for in-hospital 90-day all-cause mortality. Variables with a *P* value of < 0.2 in the univariate analysis were included in the multivariate models. The period from admission to the time of death was evaluated using the Kaplan–Meier survival analysis and log-rank test. All *P* values were two tailed, and a *P* value of < 0.05 was considered significant. All statistical analyses were performed using SPSS software (version 26.0; Statistical Package for the Social Sciences, IBM Corporation, Armonk, NY, USA).

## Results

### Baseline characteristics

A total of 178 patients were included according to the inclusion and exclusion criteria; their baseline characteristics are shown in Table 1. Twenty patients (11.2%) died within 90 days after admission (non-survivor group). The mean age of all patients was 60.0 years, and 96 (53.9%) patients were men. The proportion of organ transplantation recipients was significantly lower in the non-survivor group (5.0% vs. 25.9%, *P* = 0.048). No significant differences were observed in the incidence of other underlying diseases, diabetes mellitus, and chronic kidney disease between the two groups. The non-survivor group had a higher rate of steroid use prior to the diagnosis of PJP (35.0% vs. 12.7%, *P* = 0.016). CRP level was significantly higher in the non-survivor group (median 19.4 ± 8.6 vs. 11.4 ± 8.6 mg/dL, *P* < 0.001). However, no significant differences were found in the albumin, LD, procalcitonin, and β-d-glucan (BDG) levels between the two groups. Further, no significant differences were found in the absolute neutrophil count (ANC) and percentage of neutropenia (ANC < 1500/μL) within 6 months prior to admission.

**Table 1:** Comparison of baseline characteristics between survivors and non-survivors

	Total (n = 178)	Survivors (n = 158)	Non-survivors (n = 20)	*P* value
Age, years	60.0 ± 11.8	59.6 ± 11.1	62.9 ± 16.6	0.407
Male sex	96 (53.9)	89 (56.3)	7 (35.0)	0.095
Ever smoker	66 (37.1)	60 (38.0)	6 (30.0)	0.625
*Underlying disease*				
Hematological malignancy	58 (32.6)	53 (33.5)	5 (25.0)	0.467
Solid cancer	70 (39.3)	59 (37.3)	11 (55.0)	0.149
Organ transplantation*	42 (23.6)	41 (25.9)	1 (5.0)	0.048
Autoimmune disease	31 (17.4)	27 (17.1)	4 (20.0)	0.756
Diabetes mellitus	45 (25.3)	40 (25.3)	5 (25.0)	> 0.999
Chronic kidney disease	30 (16.9)	27 (17.1)	3 (15.0)	> 0.999
Previous steroid use**	27 (15.2)	20 (12.7)	7 (35.0)	0.016
Prophylaxis with TMP/SMX	9 (5.1)	6 (3.8)	3 (15.0)	0.066
*Initial laboratory tests*				
Albumin, g/dL (n = 177)	2.4 ± 0.5	2.4 ± 0.5	2.2 ± 0.4	0.079
C-reactive protein, mg/dL (n = 177)	12.3 ± 8.9	11.4 ± 8.6	19.4 ± 8.6	< 0.001
Lactate dehydrogenase, IU/L (n = 153), median [IQR]	375.0 [271.0–474.5]	364.0 [268.8–470.5]	427.0 [346.0–584.0]	0.069
Procalcitonin, ng/mL (n = 126), median [IQR]	0.13 [0.06–0.41]	0.13 [0.05–0.41]	0.16 [0.06–1.04]	0.593
β-D-glucan, pg/mL (n = 132), median [IQR]	72.8 [0.0–301.6]	55.4 [0.0–314.9]	98.5 [0.0–262.4]	0.801
Absolute neutrophil count, cells/μL, median [IQR]	2775.0 [1547.5–5172.5]	2775.0 [1605.0–4947.5]	2960.0 [822.5–6767.5]	0.924
Neutropenia within 6 months before admission	81 (45.5)	74 (46.8)	7 (35.0)	0.350

Data are expressed as mean ± standard deviation or number (%) unless otherwise indicated

The Fisher’s exact test was performed to analyze the variable with a value of less than 5

TMP/SMX, trimethoprim/sulfamethoxazole; IQR, interquartile range

*Organ transplantation includes 25 solid organ transplantations and 17 hematopoietic stem cell transplantations

** ≥ 20 mg/day prednisolone and ≥ 4 weeks

The baseline characteristics of the non-severe and severe PJP groups are shown in Table 2. A total of 117 patients (65.7%) were include in the severe PJP group. No differences were found in the incidence of underlying diseases (including organ transplantation), diabetes mellitus, and chronic kidney disease between the two groups. The severe PJP group had significantly lower albumin (2.3 ± 0.4 vs. 2.6 ± 0.6 g/dL, *P* < 0.001), higher CRP (14.7 ± 9.0 vs. 7.6 ± 6.6 mg/dL, *P* < 0.001), and higher LD (median 407.0, IQR 296.0–532.0 vs. 325.0, IQR 245.8–407.3 IU/L, *P* = 0.005) levels than the non-severe PJP group. However, no differences were found in the procalcitonin levels, BDG levels, ANC, and percentage of neutropenia within 6 months prior to admission between the two groups.

**Table 2:** Comparison of baseline characteristics between the non-severe PJP and severe PJP groups

	Total (n = 178)	Non-severe PJP (n = 61)	Severe PJP (n = 117)	*P* value
Age, years	60.0 ± 11.8	57.7 ± 11.7	61.2 ± 11.7	0.066
Male sex	96 (53.9)	33 (54.1)	63 (53.8)	> 0.999
Ever smoker	66 (37.1)	22 (36.1)	44 (37.6)	0.871
*Underlying disease*				
Hematological malignancy	58 (32.6)	23 (37.7)	35 (29.9)	0.315
Solid cancer	70 (39.3)	18 (29.5)	52 (44.4)	0.075
Organ transplantation*	42 (23.6)	17 (27.9)	25 (21.4)	0.356
Autoimmune disease	31 (17.4)	8 (13.1)	23 (19.7)	0.306
Diabetes mellitus	45 (25.3)	16 (26.2)	29 (24.8)	0.857
Chronic kidney disease	30 (16.9)	8 (13.1)	22 (18.8)	0.403
Previous steroid use**	27 (15.2)	6 (9.8)	21 (17.9)	0.189
Prophylaxis with TMP/SMX	9 (5.1)	3 (4.9)	6 (5.1)	> 0.999
*Initial laboratory tests*				
Albumin, g/dL (n = 177)	2.4 ± 0.5	2.6 ± 0.6	2.3 ± 0.4	< 0.001
C-reactive protein, mg/dL (n = 177)	12.3 ± 8.9	7.6 ± 6.6	14.7 ± 9.0	< 0.001
Lactate dehydrogenase, IU/L (n = 153), median [IQR]	375.0 [271.0–474.5]	325.0 [245.8–407.3]	407.0 [296.0–532.0]	0.005
Procalcitonin, ng/mL (n = 126), median [IQR]	0.13 [0.06–0.41]	0.09 [0.00–0.50]	0.13 [0.06–0.44]	0.149
β-D-glucan, pg/mL (n = 132), median [IQR]	72.8 [0.0–301.6]	38.9 [0.0–269.2]	94.2 [0.0–308.4]	0.384
Absolute neutrophil counts, cells/μL, median [IQR]	2775.0 [1547.5–5172.5]	2480.0 [1570.0–3920.0]	3110.0 [1490.0–6085.0]	0.167
Neutropenia within 6 months before admission	81 (45.5)	30 (49.2)	51 (43.6)	0.527

Data are expressed as mean ± standard deviation or number (%) unless otherwise indicated

The Fisher’s exact test was performed to analyze the variable with a value of less than 5

PJP, *Pneumocystis jirovecii* pneumonia; TMP/SMX, trimethoprim/sulfamethoxazole; IQR, interquartile range

*Organ transplantation includes 25 solid organ transplantations and 17 hematopoietic stem cell transplantations

** ≥ 20 mg/day prednisolone and ≥ 4 weeks

### BAL fluid analysis

A comparison of the BAL fluid cellular properties and PCR CT values between non-survivors and survivors is shown in Table 3. Six patients were excluded from the cellular analysis because their BAL fluid specimens were inadequate for differential cell count. The neutrophil count was significantly higher (median 22.0, IQR 2.0–46.0 vs. median 6.0, IQR 2.0–18.0%, *P* = 0.044), while the lymphocyte count was significantly lower (median 24.0, IQR 7.0–37.0 vs. median 41.0, IQR 22.5–60.5%, *P* = 0.001) in non-survivors than in survivors. Meanwhile, no difference was observed in the eosinophil and alveolar macrophage counts and the PCR CT value between the two groups.

**Table 3:** Comparison of BAL fluid cellular profile and PCR CT value in patients with PJP

	Total (n = 172)	Survivor (n = 153)	Non-survivor (n = 19)	*P* value
Nucleated cell count, cells/μL	311.0 [214.5–563.0]	311.0 [208.5–543.5]	343.0 [240.0–635.0]	0.392
Neutrophil count, %	7.0 [2.0–21.8]	6.0 [2.0–18.0]	22.0 [2.0–46.0]	0.044
Neutrophil count, cells/μL	17.3 [5.3–83.8]	16.2 [5.1–75.3]	55.9 [10.5–268.6]	0.060
Lymphocyte count, %	39.5 [20.0–60.0]	41.0 [22.5–60.5]	24.0 [7.0–37.0]	0.001
Lymphocyte count, cells/μL	113.0 [43.6–211.5]	124.0 [51.3–227.3]	71.0 [24.2–136.6]	0.068
Eosinophil count, %	0.0 [0.0–2.0]	0.0 [0.0–2.0]	0.0 [0.0–2.0]	0.587
Eosinophil count, cells/μL	0.0 [0.0–6.5]	0.0 [0.0–7.5]	0.0 [0.0–5.0]	0.408
Macrophage count, %	40.0 [24.0–57.8]	37.0 [23.0–57.0]	48.0 [40.0–60.0]	0.087
Macrophage count, cells/μL	123.2 [62.7–222.1]	118.4 [60.2–203.9]	223.0 [112.4–292.6]	0.025
CT value in PCR (n = 178), mean ± SD	28.2 ± 4.5	28.1 ± 4.5	29.1 ± 4.1	0.368

Data are expressed as median [interquartile range] unless otherwise indicated

BAL, bronchoalveolar lavage; CT, cycle threshold; PCR, polymerase chain reaction; PJP, *Pneumocystis jirovecii* pneumonia; SD, standard deviation

### Disease severity and treatment modalities

The disease severity and treatment modalities of the study patients are shown in Table 4. The non-survivor group had significantly more severe disease, lower PaO_2_ in room air, lower PF ratio (ratio of PaO_2_ [in mmHg] to fractional inspired oxygen [expressed as a fraction]), and higher (A−a)DO_2_ than those of the survivor group. Moreover, the non-survivors had shorter treatment duration, more second-line treatments, and a higher frequency of intensive care unit stay and mechanical ventilation use. However, no difference was observed in the proportion of steroid use and steroid duration between the two groups. The incidence of coincidental infections was also compared. The incidence rates of bacterial, viral, and total co-infections were not significantly different between the two groups. Only fungal infection showed a statistical difference (*P* = 0.011); however, the number of patients was relatively small (five patients) (Additional file 1: Table S1).

**Table 4:** Comparison of disease severity and treatment modalities in patients with PJP

	Total (n = 178)	Survivor (n = 158)	Non-survivor (n = 20)	*P* value
*PJP severity*				
Severe disease	117 (65.7)	97 (61.4)	20 (100.0)	0.001
PaO_2_ in room air, mmHg (n = 98), median [IQR]	65.8 [59.1–81.5]	68.7 [59.9–84.7]	52.0 [45.9–62.6]	0.003
PF ratio (n = 150)	309.5 ± 127.3	320.0 ± 129.4	241.2 ± 88.0	0.010
(A-a)DO_2_, mmHg (n = 150), median [IQR]	50.6 [35.2–89.0]	48.4 [31.9–73.0]	80.9 [53.1–134.3]	0.002
Treatment duration, days, median [IQR]	16.0 [14.0–21.0]	16.0 [14.0–21.0]	13.0 [6.8–20.0]	0.011
Second-line treatment*	30 (16.9)	23 (14.6)	7 (35.0)	0.030
Steroid use	104 (58.4)	90 (57.0)	14 (70.0)	0.339
Steroid duration, days (n = 104), median [IQR]	8.0 [0.0–16.25]	9.0 [0.0–17.0]	5.5 [0.0–13.0]	0.982
Starting steroid dose per day, mg** (n = 104), median [IQR]	75.0 [40.0–80.0]	80.0 [45.0–80.0]	40.0 [40.0–80.0]	0.061
*Starting type of steroid (n = 104)*				> 0.999
IV methylprednisolone	84 (80.8)	72 (80.0)	12 (85.7)	
Oral prednisolone	10 (9.6)	10 (11.1)	0 (0.0)	
Oral methylprednisolone	4 (3.8)	3 (3.3)	1 (7.1)	
IV hydrocortisone	6 (5.8)	5 (5.6)	1 (7.1)	
ICU stay	26 (14.6)	18 (11.4)	8 (40.0)	0.003
Mechanical ventilation	24 (13.5)	18 (11.4)	6 (30.0)	0.034

Data are expressed as mean ± standard deviation or number (%) unless otherwise indicated

The Fisher’s exact test was performed to analyze the variable with a value of less than 5

PJP, *Pneumocystis jirovecii* pneumonia; PaO_2_, partial pressure of arterial oxygen; IQR, interquartile range; PF ratio, ratio of arterial oxygen partial pressure (in mmHg) to fractional inspired oxygen (expressed as a fraction); (A-a)DO_2_, alveolar–arterial oxygen pressure gradient; IV, intravenous; ICU, intensive care unit

*Second-line treatment consists of clindamycin and primaquine

**Prednisolone equivalent dose

### Risk factors for 90-day all-cause mortalities

**Fig. 1:**
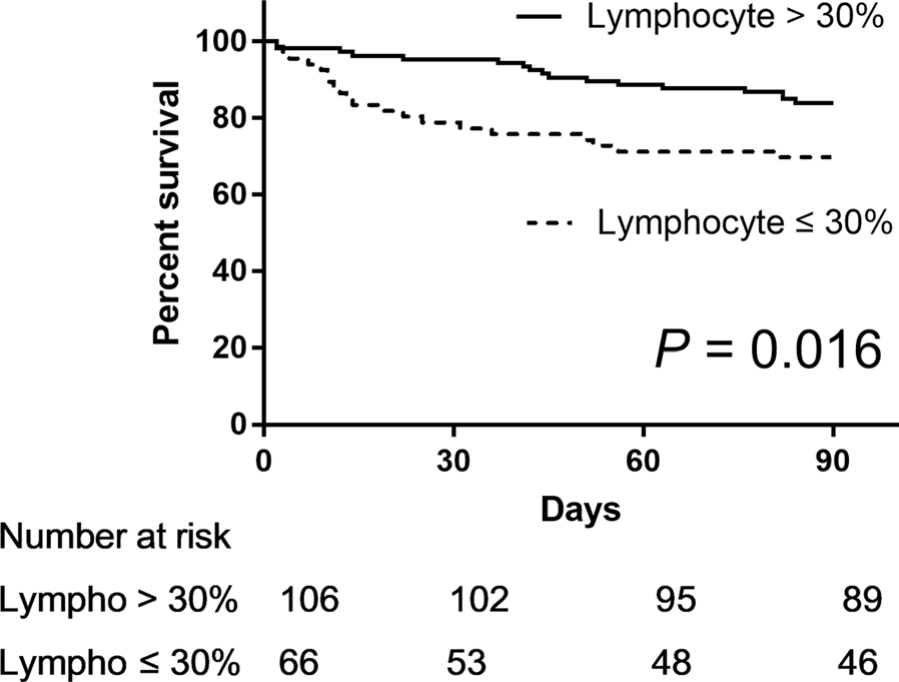
Survival curves of patients according to the lymphocyte count in bronchoalveolar lavage fluid (*P* = 0.016) Lympho, lymphocyte

**Fig. 2:**
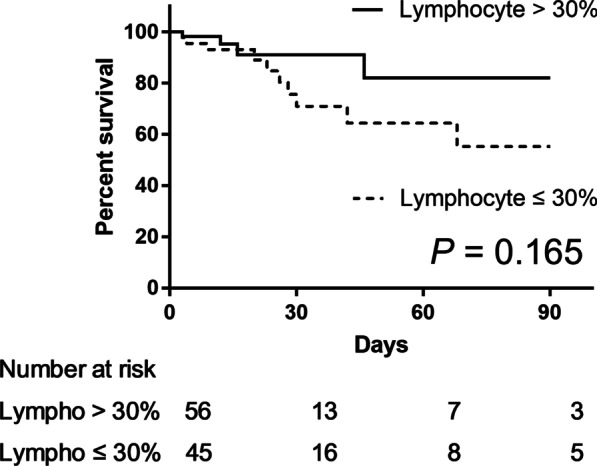
Survival curves of patients who received adjunctive steroid treatment based on the lymphocyte count in bronchoalveolar lavage fluid (*P* = 0.165) Lympho, lymphocyte

The laboratory findings and BAL fluid analysis results were analyzed to determine the risk factors for 90-day all-cause mortality. Among the BAL fluid cellular properties, lymphocyte count was selected as the surrogate marker of mortality as it showed the lowest *P* value for 90-day all-cause mortality (Table 3). Lymphocyte count (area under the curve, 0.722; *P* < 0.001) showed a higher diagnostic accuracy compared with neutrophil count (area under the curve, 0.641; *P* = 0.045) in the receiver operating characteristic curve analysis. A BAL fluid lymphocyte count of ≤ 30% was arbitrarily set as the cutoff value, with sensitivity and specificity of 68.4% and 66.0%, respectively (Youden index = 0.344). The baseline characteristics were also compared according to the BAL fluid lymphocyte count. The high lymphocyte group had significantly lower ANC and higher neutropenia percentage within 6 months prior to admission than the low lymphocyte group (Additional file 1: Table S2). In the univariate analysis, the CRP level (odds ratio [OR] 1.096, 95% confidence interval [CI] 1.042–1.154, *P* < 0.001) and BAL fluid lymphocyte count of ≤ 30% (OR 4.129, 95% CI 1.485–11.483, *P* = 0.007) were significantly associated with mortality. In the multivariate analysis, CRP level (OR 1.093, 95% CI 1.020–1.170, *P* = 0.011) and BAL fluid lymphocyte count of ≤ 30% (OR 3.353, 95% CI 1.101–10.216, *P* = 0.033) were independently associated with mortality after adjusting for albumin and LD levels (Table 5). When the post-discharge survival data were included, we found that 39 patients (21.9%) died within 90 days from the day of admission. The Kaplan–Meier curve analysis demonstrated that patients with a BAL fluid lymphocyte count of ≤ 30% had a significantly higher mortality risk than those with a BAL fluid lymphocyte count of > 30% (*P* = 0.016, Fig. 1). The Kaplan–Meier curve analysis demonstrated that among patients who received adjunctive steroid treatment, those with BAL fluid lymphocyte count of ≤ 30% tended to have higher mortality risk than those with BAL fluid lymphocyte count of > 30%; however, the difference was not significant (*P* = 0.165, Fig. 2).

**Table 5:** Risk factors for 90-day all-cause mortality of PJP according to logistic regression

Parameter	Odds ratio	95% Confidence interval	*P* value
Univariate analysis			
*Initial laboratory tests*			
Albumin, g/dL	0.394	0.138–1.123	0.081
C-reactive protein, mg/dL	1.096	1.042–1.154	< 0.001
Lactate dehydrogenase, IU/L	1.001	1.000–1.002	0.079
Procalcitonin, ng/mL	1.018	0.969–1.069	0.475
β-D-glucan, pg/mL	0.999	0.997–1.001	0.294
Absolute neutrophil count, cells/μL	1.000	1.000–1.000	0.793
*BAL fluid analysis*			
Lymphocyte count ≤ 30%	4.129	1.485–11.483	0.007
CT value in PCR	1.054	0.941–1.180	0.367
*Multivariate analysis*			
Albumin, g/dL	1.257	0.273–5.797	0.769
C-reactive protein, mg/dL	1.093	1.020–1.170	0.011
Lactate dehydrogenase, IU/L	1.001	1.000–1.002	0.063
Lymphocyte count ≤ 30%	3.353	1.101–10.216	0.033

PJP, *Pneumocystis jirovecii* pneumonia; BAL, bronchoalveolar lavage; CT, cycle threshold; PCR, polymerase chain reaction

## Discussion

The cellular profiles of BAL fluid obtained in 172 non-HIV-infected patients with PJP were analyzed. To our knowledge, this was the largest study to evaluate the prognostic value of BAL fluid analysis in non-HIV-infected patients with PJP. The BAL fluid lymphocyte count was significantly decreased in the non-survivor group. Moreover, a BAL fluid lymphocyte count of ≤ 30% was significantly associated with 90-day all-cause mortality in the multivariate analysis. These findings suggest that the BAL fluid lymphocyte count can be a useful prognostic marker of mortality in non-HIV-infected patients with PJP.

In 1980–1990, several studies reported the relationship between BAL fluid neutrophilia and mortality in HIV-infected patients with PJP [18–20]. Recently, BAL fluid neutrophilia was also reported to be associated with mortality in non-HIV-infected patients with PJP. Tamai et al. showed that a BAL fluid neutrophil count of ≥ 31% was significantly associated with in-hospital mortality in non-HIV patients with PJP [21]. Lee et al. suggested that more severe BAL fluid neutrophilia was associated with higher 30-day mortality and 60-day mortality in non-HIV-infected patients with PJP [11]. In our study, the BAL fluid neutrophil count was significantly increased in non-survivors. These findings are consistent with the results of previous studies, which suggested that neutrophilic lung inflammation in PJP may result in diffuse alveolar damage, impaired gas exchange, respiratory failure, and death [1].

However, in our study, the BAL fluid lymphocyte count was a more meaningful prognostic factor compared with the neutrophil count. The activity of CD4 + lymphocytes is important in the host defense against *P. jirovecii*, and low blood lymphocyte count, especially CD4 + lymphocytes, is associated with poor prognosis in HIV-infected patients with PJP [1]. The risk of *P. jirovecii* infection increases in patients with a CD4 + lymphocyte count of < 200 cells/μL [1], and HIV-infected patients with a CD4 + lymphocyte count of < 200 cells/μL should receive primary prophylaxis for PJP [22]. Recently, non-HIV-infected patients with decreased blood CD4 + lymphocyte counts had an increased risk of developing PJP [23, 24]. However, a few studies have investigated the relationship between BAL fluid lymphocytes and PJP prognosis. Kim et al. reported that a BAL fluid lymphocyte count of ≤ 45% was associated with failure of trimethoprim/sulfamethoxazole as first-line therapy [12]. Gaborit et al. reported that a BAL fluid cellular profile consistent with alveolitis (lymphocyte count of > 10%, neutrophil count of > 5%, and presence of activated macrophages) was associated with less severe PJP and lower 90-day mortality regardless of HIV infection status [25]. Li et al. reported that low lymphocyte count and low CD3+, CD4+, and CD8 + lymphocyte counts in BAL fluid were predictors of mortality in non-HIV-infected patients with PJP [26]. In this study, a BAL fluid lymphocyte count of ≤ 30% was significantly associated with 90-day all-cause mortality. These results suggest that alveolar lymphocytes may have an important role in the prognosis of PJP.

The survival benefit of adjunctive steroid treatment is well established in patients with HIV infection and severe PJP [1, 5]; however, the role of adjunctive corticosteroid therapy has not been validated in non-HIV patients with PJP [27]. Adjunctive corticosteroid therapy might be beneficial in non-HIV patients with PJP [28–30]. BAL fluid cell analysis has been widely used to diagnose and manage interstitial lung disease. Non-smoking healthy adults show the following BAL cellular pattern: lymphocyte count of 10%–15%, neutrophil count of ≤ 3%, eosinophil count of ≤ 1%, and alveolar macrophage count of > 85%. A lymphocyte differential count of ≥ 15% suggests hypersensitivity pneumonitis, non-specific interstitial pneumonia, drug-induced pneumonitis, or organizing pneumonia (OP) [31]. Particularly, OP rapidly responds to corticosteroid treatment and can completely remit with a clearing of radiographic abnormalities, resolution of clinical symptoms, and restoration of normal lung function [32]. Moreover, OP is a non-specific lung injury response associated with various bacterial, viral, parasitic, and fungal infections [33]. Hirasawa et al. reported that a BAL fluid lymphocyte count of ≥ 20% was significantly associated with increased survival in patients with acute respiratory failure [34]. In our study, the subgroup analysis of patients with adjunctive steroid treatment showed that the low lymphocyte group tended to have higher mortality risk, although the difference was not significant. Adjunctive steroid treatment may be effective in patients with increased BAL fluid lymphocyte count due to secondary OP. Further studies with a larger sample size are warranted to confirm the efficacy of adjunctive steroid treatment in non-HIV patients with PJP.

Some previous studies demonstrated the association between fungal burden and prognosis in patients with PJP using the BAL fluid PCR CT value or serum BDG level as surrogate markers of fungal burden. Liu et al. reported that non-survivors had a lower CT value compared with the survivors among non-HIV patients with PJP [9]. Choi et al. reported that PCR-negative conversion predicted the survival in non-HIV patients with PJP and acute respiratory failure [13]. Tamai et al. reported that non-survivors had significantly higher serum BDG levels than survivors [21]. However, our data demonstrated that both PCR CT value and serum BDG level were not significantly different between survivors and non-survivors, thus implying that inappropriate host reactions may have a more important role in PJP prognosis compared with the fungal burden.

This study has some limitations. It was a single-center, retrospective, non-randomized study. Only patients diagnosed with PJP using BAL fluid PCR were included; therefore, our study participants may not represent all patients with PJP. However, this study aimed to determine the role of BAL in non-HIV patients with PJP. The BAL lymphocyte subset was not analyzed in most patients, and only the total lymphocyte count in BAL fluid was measured. Hence, further studies on the role of CD4 + lymphocytes in BAL fluid are needed. The underlying conditions may have affected the treatment outcomes of PJP; however, this was difficult to correct owing to the retrospective nature of the study.

## Conclusions

This real-world population analysis suggests that a low lymphocyte count in BAL fluid predicts mortality and can be a useful prognostic marker in non-HIV-infected patients with PJP.

## Supplementary Information

**Additional file 1**. **Table S1.** Coincidental infections in patients with PJP. **Table S2.** Comparison of baseline characteristics between the BAL fluid lymphocyte count of > 30% group and BAL fluid lymphocyte count of ≤ 30% group.

## Data Availability

The datasets used and/or analyzed during the current study are available from the corresponding author on reasonable request.

## Abbreviations

(A-a)DO_2_: Alveolar–arterial oxygen pressure gradient
ANC: Absolute neutrophil count
BAL: Bronchoalveolar lavage
BDG: β-D-glucan
CI: Confidence interval
CRP: C-reactive protein
CT: Cycle threshold
HIV: Human immunodeficiency virus
IQR: Interquartile range
LD: Lactate dehydrogenase
OP: Organizing pneumonia
OR: Odds ratio
PaO_2_: Partial pressure of arterial oxygen
PCR: Polymerase chain reaction
PF ratio: Ratio of partial pressure of arterial oxygen to fractional inspired oxygen
PJP: *Pneumocystis jirovecii* pneumonia
SD: Standard deviation
