# Supplementary material for: Prognostic implication of bronchoalveolar lavage fluid analysis in patients with Pneumocystis jirovecii pneumonia without human immunodeficiency virus infection
Source: BMC Pulm Med. 2022 Jun 26;22:251. doi: 10.1186/s12890-022-02041-8 (PMC9233854; doi:10.1186/s12890-022-02041-8)
Supplement: Supplementary file 1 — Additional file 1. Table S1. Coincidental infections in patients with PJP. Table S2. Comparison of baseline characteristics between the BAL fluid lymphocyte count of > 30% group and BAL fluid lymphocyte count of ≤ 30% group. [file 12890_2022_2041_MOESM1_ESM.docx]

**Table S1.** Coincidental infections in patients with PJP

|  | Total  (n = 178) | Survivor  (n = 158) | Non-survivor  (n = 20) | *P* value |
| --- | --- | --- | --- | --- |
| Bacteria | 11 (6.2) | 8 (5.1) | 3 (15.0) | 0.111 |
| Virus | 44 (24.7) | 37 (23.4) | 7 (35.0) | 0.276 |
| CMV | 36 (20.2) | 30 (19.0) | 6 (30.0) | 0.374 |
| Fungus | 5 (2.8) | 2 (1.3) | 3 (15.0) | 0.011 |
| Total co-infection | 52 (29.2) | 43 (27.2) | 9 (45.0) | 0.099 |

Data are expressed as number (%).

The Fisher’s exact test was performed to analyze the variable with a value of less than 5.

PJP, *Pneumocystis jirovecii* pneumonia; CMV, cytomegalovirus

**Table S2.** Comparison of baseline characteristics between the BAL fluid lymphocyte count of >30% group and BAL fluid lymphocyte count of ≤30% group

|  | Total  (n = 172) | Lymphocyte count of >30%  (n = 107) | Lymphocyte count of ≤30%  (n = 65) | *P* value |
| --- | --- | --- | --- | --- |
| Age, years | 59.9 ± 11.6 | 57.8 ± 12.2 | 63.5 ± 9.4 | 0.002 |
| Male sex | 91 (52.9) | 59 (55.1) | 32 (49.2) | 0.529 |
| Ever Smoker | 61 (35.5) | 38 (35.5) | 23 (35.4) | > 0.999 |
| Underlying disease |  |  |  |  |
| Hematological malignancy | 58 (33.7) | 43 (40.2) | 15 (23.1) | 0.030 |
| Solid cancer | 64 (37.2) | 40 (37.4) | 24 (36.9) | > 0.999 |
| Organ transplantation* | 41 (23.8) | 28 (26.2) | 13 (20.0) | 0.461 |
| Autoimmune disease | 31 (18.0) | 14 (13.1) | 17 (26.2) | 0.040 |
| Diabetes mellitus | 44 (25.6) | 28 (26.2) | 16 (24.6) | 0.859 |
| Chronic kidney disease | 29 (16.9) | 18 (16.8) | 11 (16.9) | > 0.999 |
| Previous steroid use** | 27 (15.7) | 15 (14.0) | 12 (18.5) | 0.518 |
| Prophylaxis with TMP/SMX | 9 (5.2) | 3 (2.8) | 6 (9.2) | 0.083 |
| Initial laboratory tests |  |  |  |  |
| Albumin, g/dL (n = 171) | 2.4 ± 0.5 | 2.4 ± 0.5 | 2.4 ± 0.5 | 0.389 |
| C-reactive protein, mg/dL (n = 171) | 12.3 ± 9.0 | 11.0 ± 8.8 | 14.2 ± 9.2 | 0.026 |
| Lactate dehydrogenase, IU/L (n = 149), median [IQR] | 375.0 [271.0–474.5] | 373.5 [262.5–467–0] | 384.0 [292.0–483.0] | 0.361 |
| Procalcitonin, ng/mL (n = 122), median [IQR] | 0.13 [0.06–0.41] | 0.11 [0.05–0.18] | 0.19 [0.06–1.23] | 0.032 |
| β-D-glucan, pg/mL (n = 127), median [IQR] | 65.2 [0.0–294.0] | 58.8 [0.0–242.9] | 81.2 [0.0–314.9] | 0.620 |
| Absolute neutrophil counts, cells/μL, median [IQR] | 2775.0 [1442.5–5177.5] | 2470.0 [1200.0–3760.0] | 4020.0 [1910.0–7115.0] | 0.002 |
| Neutropenia within 6 months before admission | 80 (46.5) | 60 (56.1) | 20 (30.8) | 0.002 |

Data are expressed as mean ± standard deviation or number (%) unless otherwise indicated.

The Fisher’s exact test was performed to analyze the variable with a value of less than 5.

*Organ transplantation includes 25 solid organ transplantations and 17 hematopoietic stem cell transplantations.

** ≥20 mg/day prednisolone and ≥4 weeks

BAL, bronchoalveolar lavage; TMP/SMX, trimethoprim/sulfamethoxazole; IQR, interquartile range
